# Minimally invasive solutions for ischiofemoral impingement: case analysis and evolving surgical strategies

**DOI:** 10.1093/jhps/hnaf010

**Published:** 2025-02-12

**Authors:** Inês Palma, Afonso Nave, Tiago Torres, Ana Luísa Neto, José Campos Martins, António Seco

**Affiliations:** Orthopaedic Department, Hospital Ortopédico de Sant’Ana, Rua de Benguela, Parede 2779-501, Portugal; Orthopaedic Department, Hospital São Francisco Xavier, Estrada do Forte do Alto do Duque, Lisboa 1449-005, Portugal; Orthopaedic Department, Hospital Ortopédico de Sant’Ana, Rua de Benguela, Parede 2779-501, Portugal; Orthopaedic Department, Hospital CUF Sintra, Avenida Raúl Solnado nº8, Sintra 2710-204 Sintra, Portugal; Orthopaedic Department, Hospital CUF Sintra, Avenida Raúl Solnado nº8, Sintra 2710-204 Sintra, Portugal; Orthopaedic Department, Hospital CUF Sintra, Avenida Raúl Solnado nº8, Sintra 2710-204 Sintra, Portugal

## Abstract

Variations in the femoral version are increasingly recognized as a cause of coxalgia due to impingement or instability. The true prevalence of these variations remains unknown. The authors report a case of bilateral ischiofemoral impingement (IFI) caused by excessive femoral anteversion treated with a subtrochanteric femoral derotational osteotomy and long intramedullary nailing. We report the case of a 22-year-old female patient with deep left hip pain, consistent with IFI, refractory to conservative treatment. Imaging revealed an ischiofemoral space (IFS) of 16 mm and a femoral anteversion of 34°. A subtrochanteric femoral derotational osteotomy stabilized with a long intramedullary nail was performed. At 18 months, the same surgical procedure was performed due to similar symptoms on the right side, with an IFS of 14 mm and femoral anteversion of 35°. Joint mobility was restored bilaterally, and the symptoms were resolved. Lesser trochanter resection has been reported as a surgical option for IFI in small case series. While conventional subtrochanteric femoral derotational osteotomy with plate fixation shows good functional outcomes, pseudarthrosis remains a concern. The authors’ minimally invasive technique using the backstroke technique for osteotomy site compression helps prevent this complication while preserving the iliopsoas insertion. In this case study, a subtrochanteric femoral derotational osteotomy effectively treated bilateral IFI associated with increased femoral anteversion. The patient achieved excellent clinical outcomes with complete symptom resolution following successful consolidation of both osteotomies, though subsequent implant removal was performed to facilitate potential future surgeries.

## Introduction

Impingement syndromes are among the various causes of coxalgia. Ischiofemoral impingement (IFI) [1] is defined by a reduction in the ischiofemoral space (IFS) and quadratus femoris space (QFS), resulting in abnormal contact between the lesser trochanter and the ischial tuberosity and consequent compression of the quadratus femoris muscle.

Johnson [1] provided the initial description of this extra-articular impingement in 1977, citing three cases following hip arthroplasty. IFI is a dynamic condition that has only recently been identified as the aetiology of pain in patients without a history of trauma or surgery [1–3].

The reduction of the IFS can result from congenital factors such as coxa valga, deep hip socket, and Legg–Calve–Perthes sequelae; acquired factors such as abductor insufficiency, fractures of the lesser trochanter, or ischial tuberosity [3]; and iatrogenic factors, posthip arthroplasty [1].

Excessive femoral neck anteversion (>30°) has recently been recognized as a cause of extra-articular hip impingement [4]. This condition leads to a decrease in the abductor’s lever arm, ipsilateral knee pain, and in-toeing gait [5]. Bone impingement results in increased internal rotation and decreased external rotation in hip extension [5, 6]. These rotational deformities can occur independently or in conjunction with dysplastic changes in the hip.

Patients with IFI are predominantly female, with an average age of 50 years and a bilateral occurrence rate between 25% and 40% [7]. Deep gluteal pain is the primary symptom, aggravated by stair climbing or brisk walking. The pain is reproducible on physical examination with the IFI test (decreased external rotation in full extension) and the FABER test (combination of flexion, abduction, and external rotation) [4].

Given the multitude of predisposing conditions for the development of IFI, coupled with the rarity of the condition, a thorough clinical study confirmed by complementary exams is essential: anteroposterior (AP) and hip profile radiographs, Computed Tomography scan (CT scan)s, and Magnetic Resonance Imaging (MRI) [7, 8]. In MRI, considered the gold standard examination, the diagnosis is characterized by the presence of edema and/or fatty involution or rupture of the quadratus femoris muscle, along with a decrease in the IFS and QFS [8]. There is significant variation in these spaces’ absolute values [18–26 mm] in asymptomatic patients with IFI [8]. Singer *et al*. [2] found a strong correlation between the reduction of the IFS and QFS, quadratus femoris muscle edema, and deep gluteal pain. However, reducing these spaces alone is insufficient for diagnosis; thus, the gold standard entails a combination of positive clinical examination and imaging findings. The insertion of the psoas muscle on the lesser trochanter and the hamstrings on the ischial tuberosity can also reveal signal alterations in MRI [3, 7, 8].

Despite the significant role of femoral rotational deformity in the pathophysiology of IFI, the literature regarding treatment options and their respective outcomes remains scarce. Various hip-preserving techniques have been described in small case series [9, 10].

The authors present a case of a patient undergoing bilateral subtrochanteric derotational femoral osteotomy, stabilized with an intramedullary nail.

## Description of the clinical case

The clinical case concerns a 22-year-old female patient with a personal history of hypermobility syndrome (Beighton score 5 out of 9). She sought outpatient care for left hip pain that had persisted for 2 years. The deep gluteal pain had mechanical characteristics without radiating pain, paraesthesia, or leg weakness.

**Figure 1.**
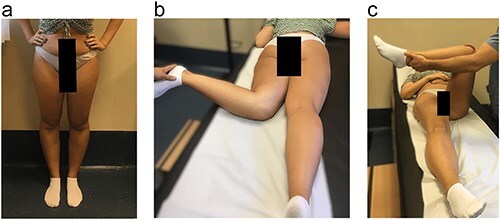
(a) Clinical examination. (b) Left hip with 90° internal rotation. (c) FABER test positive.

On physical examination (Fig. 1), she exhibited pain in the quadratus femoris and ischial region. Notably, she displayed an in-toeing gait pattern, a positive FABER (Flexion, Abduction and External Rotation) test, and a positive IFI test, with negative FADIR (flexion, adduction, internal rotation) and DIRI (Dynamic internal rotatory impingement) tests. The Harris Hip Score (HHS) assessment resulted in 45 points, the Hip Disability and Osteoarthritis Outcome Score (HOOS) of 67.5% and the initial visual analogue scale (VAS) was 8.

**Figure 2.**
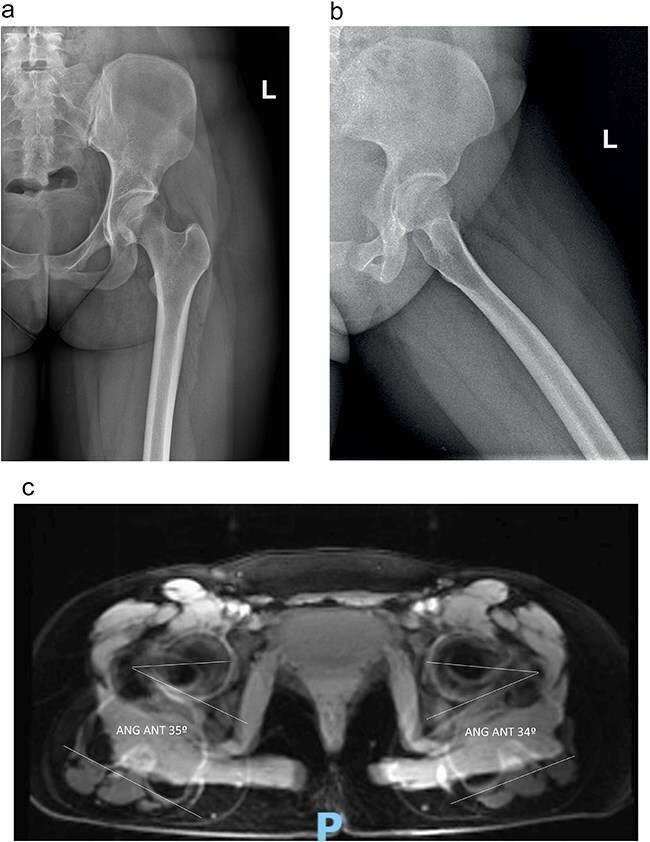
The preoperative AP (a) and lateral (b) radiographs; and the MRI scan (c) with measurement of femoral version: with high femoral version (34° left and 35° right).

**Figure 3.**
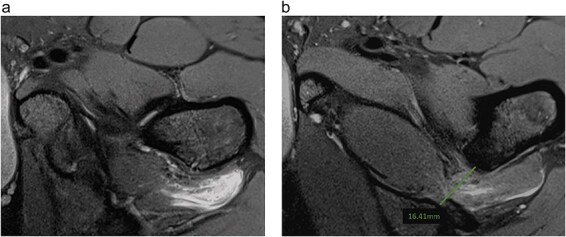
Axial T1-weighted fat-suppressed MRI images after intravenous injection of gadolinium demonstrate (a) enhancement surrounding quadratus femoris muscle (*) (b) EIF left hip measuring 16 mm.

The radiographs of pelvis in AP and frog lateral projection showed no abnormal finding. The left hip MRI, performed using a high-field magnetic resonance imaging (3 Tesla), revealed increased signal intensity of the quadratus femoris muscle with concurrent narrowing of the IFS. On axial T2-weighted fat-suppressed magnetic resonance images, there were diffuse edema and increased signal intensity within the quadratus femoris muscle bilaterally. It was compatible with IFI syndrome revealing a left IFS of 16 mm, with excessive femoral anteversion of 34° and tendinopathy of the hamstrings and adductors (Figs 2 and 3). There were aspects of sciatic nerve neuritis along the IFS (where atrophy of the quadratus femoris with fibrotic bands in this region is observed). Additionally, she presented with borderline dysplasia with a Wiberg angle of 24°, without labral tear. There were no abnormal findings, such as degenerative arthritis, femoroacetabular impingement, or osteonecrosis.

**Figure 4.**
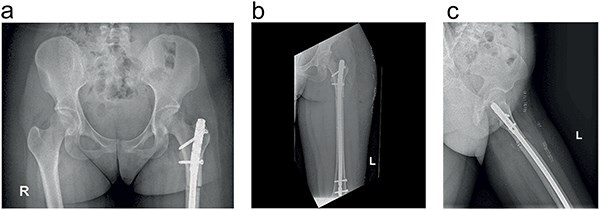
The postoperative AP (a and b) and lateral (c) radiographs after the subtrochanteric femoral osteotomy (derotation of 20°) and fixation achieved with an intramedullary nail locked proximal and distal are shown.

The patient remained refractory to conservative treatment and subsequently underwent subtrochanteric derotational osteotomy. The patient was positioned in the supine on a traction table. An intraoperative correction of 20° was achieved, confirmed by goniometer and radiographic control. Fixation was performed using an Expert LFN intramedullary nail (Synthes) with static distal locking and dynamic proximal locking. A residual postoperative diastasis between major bone fragments following intramedullary nailing of long-bone fractures is recognized as one of the major risk factors for delayed union and non-union. Axial compression of the osteotomy site was achieved using the backstroke technique, followed by oblique static proximal blocking (Fig. 4). With the hammer guide attached to the connector and insertion handle, light reverse hammer blows may be used to compress the fracture, while monitoring reduction radiographically. A shorter nail should be chosen when back-hammering is planned to prevent excessive protrusion of the nail. Furthermore, platelet-rich plasma was intraoperatively injected into the adductor and hamstring muscles to address tendinopathy as an adjunct, thereby enhancing its treatment.

Rehabilitation began on the first postoperative day, involving gait training with crutches with load as tolerated, joint mobilization, and unrestricted muscle strengthening. At 6 weeks postoperative, she had no pain complaints, experienced mild limping while walking with one crutch, and showed good progress in osteotomy consolidation. The patient remained asymptomatic, with a negative FABER test, a good gait pattern, and full hip mobility.

At 18 months of follow-up, she reported right hip pain accompanied by deep gluteal pain consistent with IFI. On physical examination, bilateral external rotation was 80°, and internal rotation was 45° on the left and 80° on the right. Regarding the nonoperated hip, there was a positive IFI test, without symptoms related to the sciatic nerve. The HHS assessment resulted in 44 points, HOOS of 69.4% and the initial VAS was 6. The right hip MRI revealed edema of the quadratus femoris, suggestive of a potential dynamic conflict in the IFS, with an IFS of 14 mm and an increased femoral neck anteversion angle close to 30°. There was mild osseous overcoverage with a global ‘pincer’ type morphological configuration, without evidence of sciatic nerve neuritis or any other abnormal findings. These observations were again consistent with IFI syndrome.

**Figure 5.**
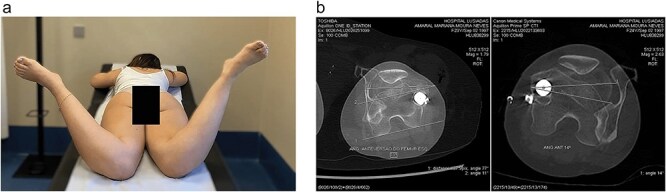
(a) Clinical examination showing hip internal rotation: 45° left and 45° right. (b) CT scans with measurement of both femoral versions correction.

The patient underwent conservative treatment with nonsteroidal anti-inflammatory drugs and rehabilitation program without resolution of the condition, leading to a surgical treatment proposal. She underwent a right femoral subtrochanteric derotational osteotomy, which proceeded without complications. An intraoperative correction of approximately 25° was achieved. At 6 weeks postoperative, she was asymptomatic with internal rotation of 45° and external rotation of 80° bilaterally (Fig. 5a). Mild limping during walking persisted until 3 months postoperatively.

**Figure 6.**
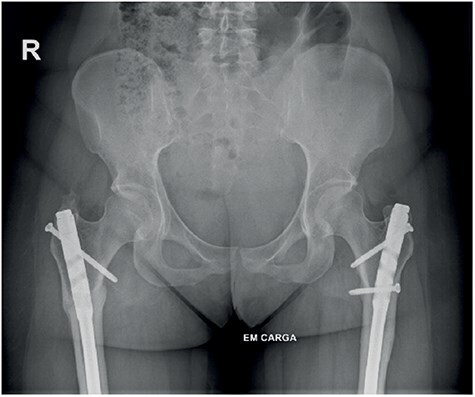
Healed femoral osteotomies.

Clinically and radiographically, the patient exhibited corrected femoral torsion (left 14°, right 11°) (Fig. 5b) and consolidation of both osteotomies with good alignment (Fig. 6). The patient maintained slight limp only at the beginning of walking during the first 6 months, with an improvement in the HHS to 100 and HOOS to 100% bilaterally and pain was decreased gradually to VAS 0, at the first year postoperatively.

Despite being asymptomatic, considering the patient’s age, we recommend the removal of the osteosynthesis material to facilitate potential future arthroplasties of the hip or knee, thereby avoiding future difficulties. Subsequently, a year and a half after the second procedure, the patient underwent bilateral removal of the osteosynthesis material during the same surgical procedure, resulting in complete resolution of the initial symptoms.

## Discussion

Our case illustrates key aspects of IFI diagnosis and management. The patient presented with classic features, including deep gluteal pain, positive IFI test [2], quadratus femoris edema on MRI, and a narrowed IFS [8]. Notably, the presence of increased femoral anteversion (34° and 35° bilaterally) reflects the emerging recognition of version’s role in IFI [4–6].

Although this patient did not report any specific sciatic nerve symptoms, the proximity of the IFS to the sciatic nerve raises the possibility that some patients may experience referred pain or nerve irritation. Differentiation of IFI from deep gluteal syndrome, which can present with similar posterior hip pain, was based on a combination of clinical tests with dynamic impingement simulation and MRI findings. However, the possibility of concomitant nerve irritation or referred pain cannot be definitively excluded without detailed neurological examination or nerve conduction studies. Further research with preoperative and postoperative neurological and electrophysiological assessment may help to better elucidate the relationship between IFI and deep gluteal syndrome and guide optimal management of these conditions.

Following failure of conservative treatment at 3 months [10], bilateral derotational osteotomies were performed. The procedures achieved femoral version correction to 14° left and 11° right, with excellent outcomes including resolution of symptoms, restored internal rotation from 80° to 45° bilaterally. Additionally, the patient demonstrated significant improvements in all Patient-Reported Outcome Measures. This experience adds to growing evidence supporting derotational osteotomy for IFI with increased femoral anteversion, demonstrating predictable improvement in hip mobility and high rates of symptom resolution [4, 9].

Recent literature shows evolving understanding of IFI, but several key aspects remain debated, including optimal diagnostic criteria, treatment algorithm, and the role of femoral version. First, while MRI findings of quadratus femoris edema and narrowed IFS are well described [7, 8], the threshold values for pathologic narrowing vary between studies. The relative importance of clinical versus imaging findings in making the diagnosis remains unclear [2, 3]. Regarding the treatment algorithm, while Suren *et al*. [10] propose starting with 3 months of conservative treatment before considering surgery, the optimal duration and components of nonoperative management are not standardized. The indications for different surgical techniques (lesser trochanter procedures versus osteotomy) continue to evolve as our understanding of underlying pathoanatomy improves [4, 9]. Lastly, the relationship between increased femoral anteversion and IFI is increasingly recognized [4–6], but specific version thresholds for considering derotational procedures versus isolated lesser trochanter surgery are not established.

The surgical options described in the limited literature include open resection of the lesser trochanter [1] and endoscopic techniques for partial [11] or complete [12] resection of the lesser trochanter. In cases associated with excessive femoral anteversion, derotational osteotomies fixed with a plate [13, 14] or a long intramedullary nail [9] are described.

The results of endoscopic lesser trochanter procedures have shown promise in several small series. Hatem *et al*. [11] reported on five patients who underwent endoscopic partial resection of one-third of the lesser trochanter through a posterior approach via the quadratus femoris. Their patients showed significant improvement with HSS increasing from 51 to 94 points and returning to sports averaging 4.4 months. Similarly, Wilson *et al*. [12] described seven successful cases of complete endoscopic resection with iliopsoas tenotomy, demonstrating maintained hip flexion strength at 1 year follow-up. In both series, diagnostic and therapeutic injections of ropivacaine and corticosteroid into the QFS and IFS were used to confirm the diagnosis preoperatively.

However, both groups emphasized important considerations for optimal outcomes with endoscopic techniques: careful patient selection with particular attention to underlying femoral version abnormalities; complete and adequate resection of the lesser trochanter when performed; confirmation of diagnosis through preoperative injections; and recognition that some patients may be better served by derotational osteotomy if significant version abnormalities exist [4, 9].

The results of arthroscopic resection of the lesser trochanter are based on small case series or individual reports. The research conducted by Hatem *et al*. [11] is notable, providing a comprehensive analysis of a 2-year follow-up involving five patients who underwent endoscopic partial resections of one-third of the lesser trochanter. This procedure was carried out via a posterior approach through the quadratus femoris. Patients showed an improvement in the HSS from 51 to 94, with a return to sports in an average of 4.4 months. Wilson *et al*. [9] published seven cases undergoing complete endoscopic resection of the lesser trochanter and iliopsoas tenotomy, with no subsequent decrease in hip flexion strength after 1 year of follow-up. In both case series, diagnostic and potentially therapeutic injections of ropivacaine and corticosteroid were administered into the QFS and IFS prior to the procedures [12]. Both studies concluded that the ineffectiveness of these endoscopic procedures can be attributed to inadequate debridement or failure to diagnose femoral version abnormalities [9, 12].

Several series have reported outcomes of femoral subtrochanteric derotational osteotomies for patients with symptomatic IFI associated with high femoral anteversion. Both lesser trochanter procedures and osteotomies appear to have distinct advantages. Endoscopic lesser trochanter procedures have been demonstrated to maintain hip flexion strength as shown by Wilson *et al*. [12], with a lesser invasive approach and good short-term outcomes in properly selected patients [11, 12]. Derotational osteotomies offer correction of underlying version abnormality as demonstrated by Lerch et al. [4] showing reduction in positive IFI test from 100% to 4%, as well as improvement in hip rotation parameters with increased external rotation from 16° to 44° [4], and option to address associated deformities like coxa valga when present [9].

Tonnis *et al*. [13] reported on seven cases of osteotomies using a plate fixation (4.5 mm DCP plate) with improvement in symptoms in 83% of cases and improved rotation parameters. Kamath *et al*. [14] described combining this approach with surgical hip dislocation, though noting a 7% delayed union rate.

Lerch *et al*. [4] conducted a retrospective study involving 25 hips (23 patients) exhibiting symptoms of IFI and excessive femoral anteversion, with a minimum follow-up of 2 years. The authors assessed pain, function, and range of motion of the hip, complications and subsequent surgeries, as well as subjective satisfaction through a questionnaire. Surgery was recommended for all cases involving patients with symptoms persisting for more than 6 months. The osteotomy was combined with surgical hip dislocation, except in one case, for the correction of femoroacetabular impingement, labral or cartilage lesions, and dynamic assessment of impingement. Positive clinical tests for IFI decreased significantly from 100% to 4%, with an increase in external rotation from 16° to 44° and a reduction in internal rotation. In 64% of cases, subsequent surgeries were related to material extraction [4].

Recently, Buly *et al*. published a study involving a total of 55 subtrochanteric derotational osteotomies fixed with an intramedullary nail, achieving excellent outcomes in 75% of cases of femoral torsion correction over a 6.5-year follow-up period. The authors highlighted a case of pseudarthrosis and one infection. In this study, 70% of patients underwent implant removal, most commonly due to complaints related to the proximal screw.

In cases of coxa valga associated with femoral version abnormalities, techniques involving varus derotational intertrochanteric osteotomies are described, resulting in abductor shortening [9]. Suren *et al*. [10] also proposed modifying this technique by incorporating lateralization and varization of the osteotomy, which is stabilized with an angular plate, particularly in cases of coxa valga and excessive femoral anteversion. This demanding surgical technique allows for the normalization of muscle lever arms. It is contraindicated in patients with genu valgum due to the consequent valgization of the anatomical axis of the femur. In this series of four patients, symptoms of IFI resolved, leading the authors to consider this technique promising.

In all case series, a decrease in internal rotation and an increase in external rotation are reported following derotational osteotomy [4, 9, 10]. With the restoration of normal hip ranges of motion, most patients’ typical pain associated with external rotation to extension is alleviated. However, postoperative complaints of limping while walking and knee pain may occur [9].

Lerch *et al*. [4] did not report any cases of delayed union or the need for revision surgery. Nonetheless, a delayed union rate of approximately 7% has been reported in subtrochanteric osteotomies [14]. Kamath *et al*. [14] recommend curettage of the osteotomy site to promote consolidation. Most authors recommend applying compression at the osteotomy site to minimize consolidation issues [4]. In the previously described case by the authors, axial compression of the osteotomy site was achieved using the backstroke technique after nail insertion, resulting in the consolidation of both osteotomies without complications. Backstroke technique enhances osteotomy consolidation by promoting direct bone healing, reduces the risk of nonunion and maintains alignment and biomechanical integrity of the limb. Additionally, the possibility of axial dynamization of the nail provides another tool to correct union delays, significantly reducing this complication.

We conclude that in the etiological study of IFI, it is crucial to identify any excessive femoral anteversion, often combined with other hip deformities, to optimize outcomes by enabling correction during the same surgical procedure. Subtrochanteric derotational femoral osteotomy is a safe and effective procedure for correcting the femoral version, with the advantage of restoring hip biomechanics and preserving the iliopsoas insertion. Unlike plate fixation, nail fixation allows for a minimally invasive approach, with a lower risk of infection and minimal dissection that promotes osteotomy consolidation [9, 10]. Alongside the reduced risk of postoperative trochanteric pain, nail fixation permits early full weight-bearing. A disadvantage of the nail entry point is the potential for injury to the abductors, which inherently have a 30% smaller lever arm [4, 15].

In all the cited case series, over two-thirds of patients undergo implant removal, with a similar percentage in both plate-fixed and nail-fixed osteotomies [4, 9, 10]. The need for hardware removal in a significant proportion of patients can be attributed to various factors, such as patient discomfort, irritation caused by the implant, or the surgeon’s preference to remove the hardware to prevent potential complications in the future, especially in younger patients who may require additional surgeries.

## Data Availability

The data underlying this article are available in the article and in its online supplementary material.
